# Mitral Valve Endocarditis Secondary to Nasal Irrigation Use in Chronic Allergic Rhinitis

**DOI:** 10.7759/cureus.28886

**Published:** 2022-09-07

**Authors:** Bradley Casey, Destinee Hua, James Barton, Bhaskar Chhetri

**Affiliations:** 1 Internal Medicine, Cape Fear Valley Medical Center, Fayetteville, USA

**Keywords:** right bundle brach block, seasonal allergies, streptococcus mitis, nasal irrigation, mitral valve endocarditis

## Abstract

*Streptococcus* *mitis* (*S. mitis) *is a common colonizer of the teeth, nasopharynx, and oropharynx. *S. mitis* has been reported in several cases of streptococcal infective endocarditis (IE). Streptococcal IE is most associated with dental procedures and diseases of the mouth. There are fewer reports of diseases of the nasopharynx leading to endocarditis secondary to nasal irrigation systems, and that is why we present a unique case of mitral valve IE secondary to nasal irrigation. We report a case of a 49-year-old African American woman with a history of chronic allergic rhinitis who presented with chest pain and subjective fevers. Transthoracic echocardiogram (TTE) failed to show valvular vegetation, but high clinical suspicion led to transesophageal echocardiogram (TEE) imaging that demonstrated a mobile echo density with a size of 5mm by 3mm attached to the atrial side of the anterior mitral valve leaflet with thickening of the anterior mitral leaflet tip as well as moderate mitral valve regurgitation. Findings on TTE were consistent with IE. The patient still has organized/nodular vegetation after three months of appropriate antibiotic therapy. We highlight how poor nasal hygiene is low on the differential for a cause of valvular endocarditis. This case will help clinicians in determining appropriate therapy for chronic allergic rhinitis. This will also help clinicians to inform patients to stop using nasal irrigation systems if epistaxis is present.

## Introduction

Infective endocarditis (IE) occurs most frequently with intravenous (IV) drug abuse, and the most isolated agents are Staphylococcus and Streptococci species [1]. *Streptococcus mitis* (*S. mitis*) is a common colonizer of the teeth, nasopharynx, and oropharynx. *S. mitis* is usually considered a commensal organism, however, has been reported in cases of streptococcal IE [2]. Oral Streptococci contain adhesive molecules that allow them to efficiently colonize different tissues in the oral cavity, and pathogenic streptococci residing in the oral cavity can eventually gain access to the bloodstream and cause systemic infections such as IE [2]. There are fewer reports of diseases of the nasopharynx leading to endocarditis. This case presentation describes the hospital course of a patient with acute on chronic allergic sinusitis managed with frequent nasal irrigation who presented with chest pain and fever. Laboratory workup was remarkable for right bundle branch block (RBBB) on electrocardiogram (EKG) and blood cultures positive for *S. mitis*. A transesophageal echocardiogram (TEE) confirmed the diagnosis of streptococcal IE. The patient lacked other common risk factors of endocarditis leading to high suspicion of etiology to be a result of repeated nasal flushing.

## Case presentation

**Figure 1:**
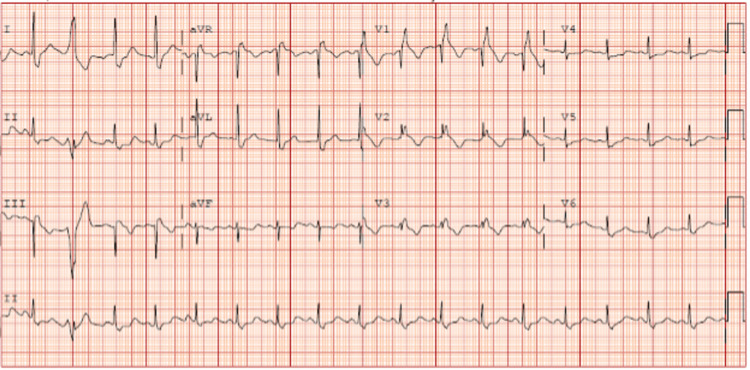
Electrocardiogram on admission showing sinus tachycardia 106 BPM, RBBB, and premature ventricular complexes

**Figure 2:**
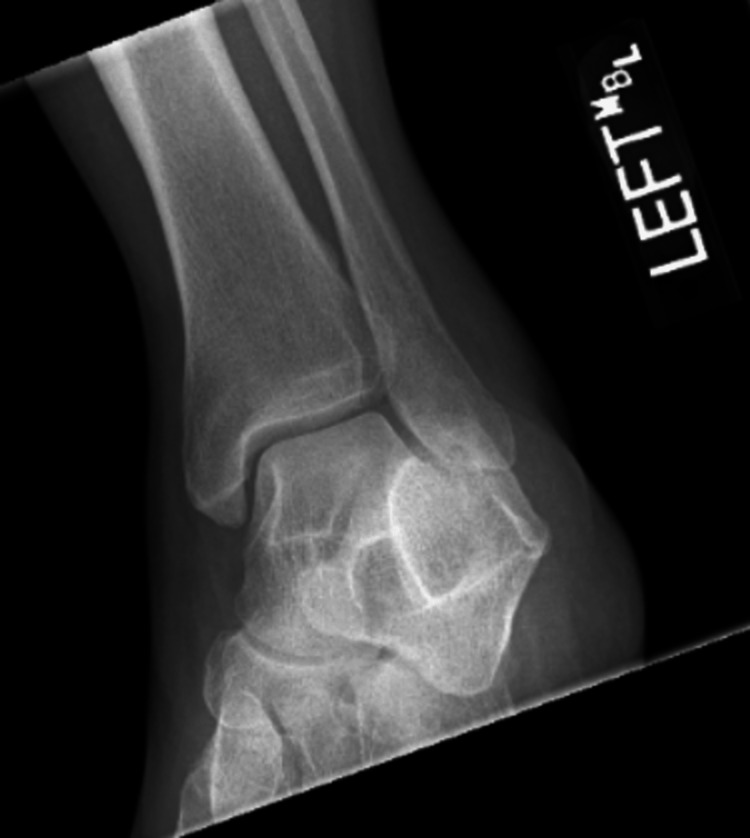
X-ray of left ankle showing soft tissue swelling and small ankle effusion

**Figure 3:**
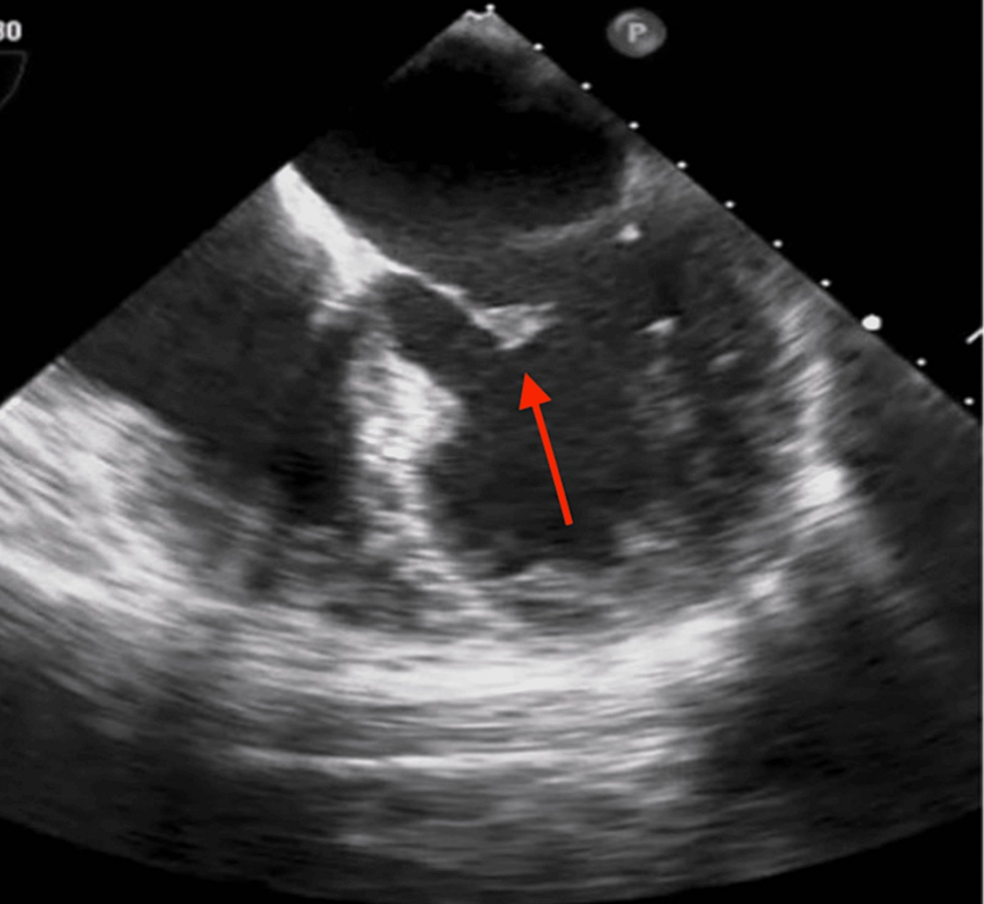
Transesophageal echocardiogram demonstrating a 5mm by 3mm anterior mitral valve vegetation

A 49-year-old African American female with a past medical history of chronic allergic rhinitis, fibroids, and normocytic anemia presented to the emergency room with chest pain for 10 days. Chest pain was described as sharp, four out of 10, mid-sternal, non-radiating, and worse with deep inspiration and movements. In addition to her primary symptoms, she had malaise, chills, fatigue, and complaining of a subjective fever for a week as well as right-sided sinus pressure with epistaxis. The patient performed nasal irrigation four times daily after the onset of nasal congestion and epistaxis. After three days of nasal irrigation, she developed worsening fatigue and subjective fevers. On presentation to the emergency room, she had intermittent febrile episodes with a maximum temperature of 103.1F, which was treated with acetaminophen. The patient remained afebrile during the hospital stay after the initiation of antibiotics. In the emergency department, she also had sinus tachycardia of 110s that improved after fluid resuscitation with lactated ringers. The patient’s electrocardiogram (EKG) (Figure 1) showed sinus tachycardia of 106, ventricular premature complexes, and RBBB. There was no previous EKG to compare to see if the bundle branch block was new. Chest x-ray showed no signs of active disease and no evidence of pneumothorax or pleural effusion. A complete blood count with differential showed leukocytosis with a white blood count of 15.5 x10^3^/µL (Normal 4.0 - 12.5 x 10^3^/µL) with an absolute neutrophil count of 12.55 x10^3^/µL (normal 1.7-7.0 x10^3^/µL). Blood biochemistry showed a c-reactive protein count of 85.2 mg/L (normal less than 3.0 mg/L), serum lactate level of 0.9 mmol/L (normal 0.5-2.0 mmol/L), and high sensitivity troponins max of 453 pg/mL (normal 2-15 pg/mL). She tested negative for COVID-19 by nasal swab and RTPCR, had an unremarkable urinalysis, and tested negative for all substances on urine drug screening. At this time, the patient was admitted to hospitalist service. Two peripheral blood cultures were obtained from two different sites before she was started on empirical antibiotic treatment with vancomycin and cefepime. The blood cultures tested positive in four out of four bottles for *S. mitis* after 24 hours. Due to concern of sinus abscess, a CT scan of sinuses with contrast was ordered and showed narrowing of the right ostiomeatal. Transthoracic echocardiogram (TTE) was unremarkable other than trace mitral valve regurgitation. On physical exam the day after her TTE, she developed a Janeway lesion on her left thumb and had swelling and pain in her left ankle. An x-ray of her left ankle was obtained, and it showed a small effusion and overlying soft tissue swelling (Figure 2). The ankle effusion and Janeway lesion were attributed to septic emboli. Due to high clinical suspicion of IE, the patient underwent a TEE, which demonstrated a mobile echo density of 5mm by 3mm attached to the atrial side of the anterior mitral valve leaflet with thickening of the anterior mitral leaflet tip as well as moderate mitral valve regurgitation (Figure 3) and (Video 1). Infectious disease was consulted once the patient’s initial blood cultures showed Streptococcus bacteremia. She was started on IV ceftriaxone and vancomycin. Once the final speciation of the peripheral blood cultures revealed *S. mitis*, vancomycin was discontinued, and she was started on IV ceftriaxone for six weeks. A PICC line was placed in her left upper arm, and she was discharged home with instructions to continue receiving daily ceftriaxone infusions at an outpatient infusion center.

**Video 1:** Mobile echo density 5mm by 3mm attached to the atrial side of the anterior mitral valve leaflet with thickening of the anterior mitral leaflet tip

**Figure 4:**
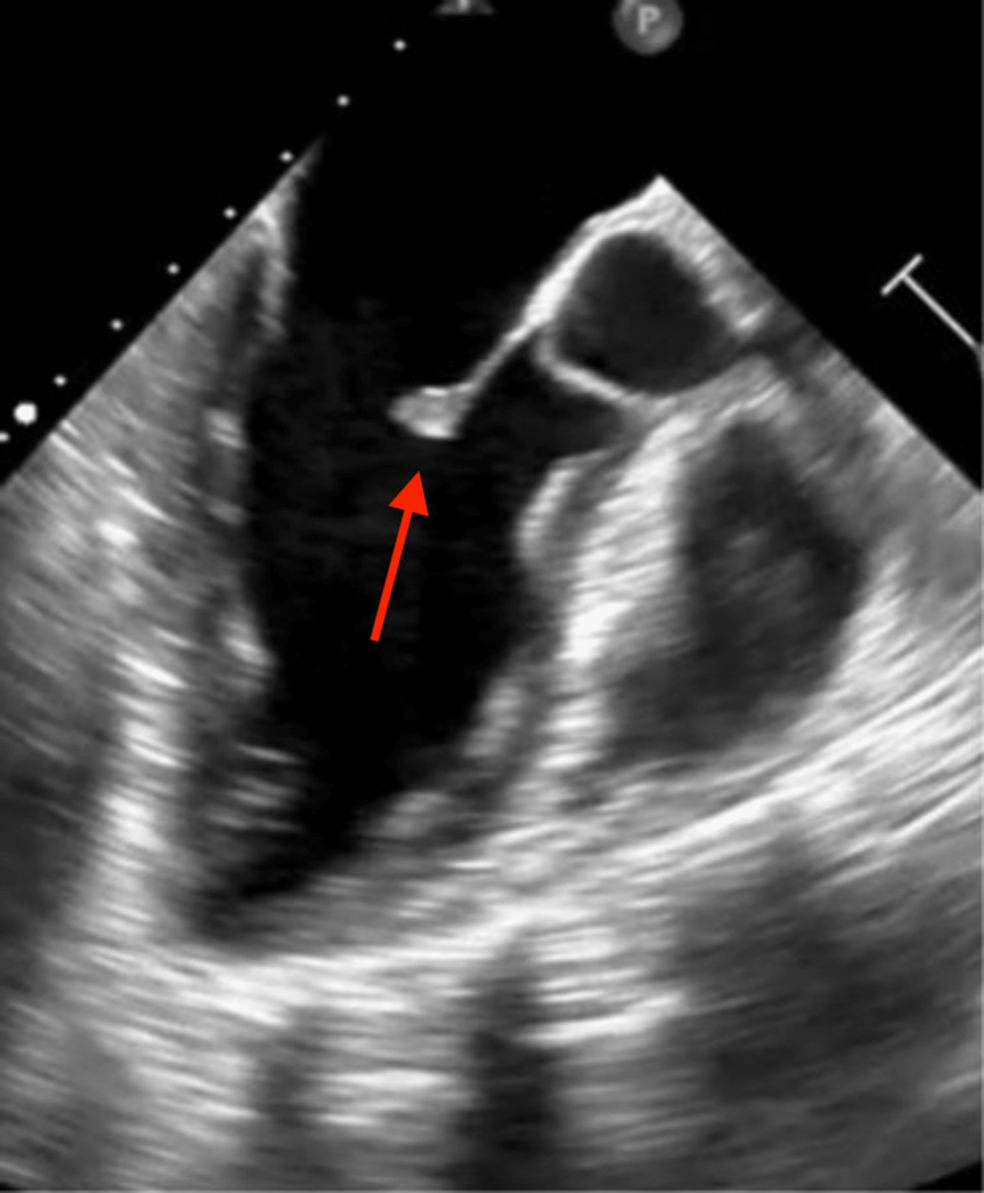
Three-month follow-up transesophageal echocardiogram demonstrating a more organized/nodular anterior mitral valve vegetation

The patient completed four weeks of intravenous ceftriaxone for Streptococcus mitral valve endocarditis. She underwent three-month follow-up TEE that showed a more organized/nodular vegetation and lacks the mobile component seen previously (Figure 4).

## Discussion

IE patients may not always present with classical signs and symptoms, so the modified Duke criteria should be applied to patients with suspected IE. Echocardiography is crucial for the diagnosis of IE. TTE is usually the initial test in most patients, due to its cost-effectiveness and low risk to the patient. TEE is recommended when the initial transthoracic echocardiography is negative in the setting of high clinical suspicion. IE has a high mortality rate, despite treatment with appropriate antibiotic therapy. IE has an in-hospital mortality rate of 15%-20% and a one-year mortality rate near 40%. The incidence of IE is 3-10 per 100,000 patient-years [1].

Streptococcal bacteria are common colonizers of the human body and are currently broken down into eight distinct groups. Of particular interest to this case study is the mitis group, the largest streptococcal group to colonize the human oral cavity with over 20 unique species. In this group, *S. mitis* is a common colonizer of the teeth, nasopharynx, and oropharynx and is usually considered a commensal organism; however, it has been implicated in many cases of IE [2]. A Danish multivariable regression study of 6,506 cases of streptococcal bloodstream infections found that the rate of IE in patients with a streptococcal bloodstream infection was 7.1% and that *S. mitis* accounted for 19.4% of these streptococcal IE cases [3]. While not the primary cause of IE, streptococcal IE is very prevalent and is commonly associated with dental procedures and diseases of the mouth, such as gingivostomatitis. In fact, people who suffer from gingivitis are more likely to develop bacteremia and subsequent IE from brushing their teeth or flossing [4]. The overall percentage of IE cases attributed to streptococcal species in the United States in 2011 was 27% while the highest percentage of IE cases came from Staphylococcal species at 40% [5].

Although dental work and poor oral hygiene have been well documented and associated with Streptococcal IE, there are fewer studies linking it to poor nasal hygiene. Streptococcal species also commonly inhabit the nasopharynx and there have been case studies implicating nasal packing for epistaxis as a nidus for bacteremia [6,7]; however, to our knowledge, there have never been any cases associating the use of nasal saline irrigation systems and streptococcal IE. Our patient endorsed having sinus pressure and episodes of epistaxis for which she used nasal irrigation wash to relieve her symptoms. She repeatedly attempted to clear her sinuses. She continued to use nasal irrigation to help moisturize her nasal passageway which led to the mixing of commensal *S. mitis* in her nasopharynx with her blood which eventually led to bacteremia and IE.

## Conclusions

While our patient did not have any risk factors for IE, it is important to consider the potential harm of recommending nasal saline irrigation for symptomatic relief of nasal congestion in patients that have artificial heart valves, congenital heart malformations, previous heart disease, or immunodeficiencies thus making them more susceptible to IE. Patients should be advised about symptoms like epistaxis, and to stop using the irrigation system if these symptoms occur. Endocarditis can be fatal, and it is important to use your clinical judgment when advising patients to use nasal irrigation for recurrent sinus congestion.
